# Supplementary material for: Genome-Wide Identification of CCD Gene Family in Six Cucurbitaceae Species and Its Expression Profiles in Melon
Source: Genes (Basel). 2022 Jan 28;13(2):262. doi: 10.3390/genes13020262 (PMC8872574; doi:10.3390/genes13020262)
Supplement: Supplementary file 1 [file genes-13-00262-s001.zip › genes-1514426-supplementary.pdf]

Supplementary material

Table S1. Specific primers of melon *CCDs* used for qRT-PCR in this studyss

Gene	Forward primer sequence（5′–3′）	Reverse primer sequence（5′–3′）
CmCCD1	GCTACACTGGCAGGAAGCAACAA	ACCAGGTCCAAGGTCATAGAGGC
CmCCD4	TCCAGCAATGGCGAGGGTCT	GAGCGAAGTTGTCGGCGAGAAT
CmCCD7	TTCCATCGCCCATCAAACTTCCTC	GTCTCGGTCCGCTGTGAAATGAA
CmCCD8	TGCGAACATAACGCCAATCCTG	GGTCCAATGCTGCCTCCAACT
CmCCDL	GCTTGGAACAATCGCCCATTTACG	CGCCTCTGATAAGTCGGTTGAAGT
CmNCED3	CGCAGACGCCAACGCCATTA	GCGGTGTCATGCACGATCCAAT
CmNCED5a	GAGACGGAGGAGGTGGTGGTAAT	GCAATCGCCAAATACGCATACCG
CmNCED5b	CGGAATCGGAGCACATGAACCT	ATCCACCTTCGCAAACCCACAG
CmNCED6	GTTCTGGCATTGCGAAGGTGGA	AAACCCTCCGTCCTCGTTCTCA
